# Opinion: “Heart Rate Variability, Health and Well-Being: A Systems Perspective” Research Topic

**DOI:** 10.3389/fpubh.2017.00246

**Published:** 2017-09-25

**Authors:** Angela J. Grippo

**Affiliations:** ^1^Department of Psychology, Northern Illinois University, DeKalb, IL, United States

**Keywords:** animal models, autonomic function, heart rate variability, prairie vole, social behavior, systems perspective, translational research

The promotion of health and well-being is a long-standing goal of scientific inquiries. The World Health Organization mission incorporates not only the absence of disease but also an integrative perspective on social, physical, and mental well-being (1). The National Institutes of Health goals are intimately focused on protecting and improving health through funding research and facilitating programs that promote public health (2). Healthy People is an example of a broadly focused, long-range strategic initiative that incorporates results from scientific inquiry into health promotion recommendations (3). Therefore, scientific strategies to understand, describe, and predict health-related issues are important steps in the process of promoting health and welfare.

The Frontiers research topic titled “Heart Rate Variability, Health, and Well-being: A Systems Perspective” is focused on the construct of heart rate variability (HRV) in the context of health and well-being. Autonomic control of peripheral functions involves a complex series of interactions, including the sympathetic and parasympathetic nervous systems as well as central processes, neuroendocrine functions, and reflex arcs. Therefore, studies involving HRV have the potential to provide insight into a wide variety of psychological and physiological processes.

Altered HRV, and its value for predicting dysfunction (or lack thereof), has contributed significantly to our understanding of several conditions [for reviews, see Ref. (4, 5)] such as stress reactivity and resilience (6, 7); emotional reactivity and personality factors (e.g., responses to stress, perception of stress, emotional memory, attention and related cognitive functions, and self-regulation processes) (8–12); emotion-related disorders (e.g., depression, anxiety, and sleep disorders) (13, 14); social behaviors (e.g., social engagement and social–emotional processes) (15–17); cardiovascular functions and risk factors (e.g., hypertension, myocardial infarction, sudden cardiac death, renal dysfunction, and diabetes) (4); and cancer (18). The study of HRV also has provided insight into the interactions of psychological and physiological processes [for review, see Ref. (15)], including stress reactivity following cardiac transplants (8); effects of antidepressants on autonomic function in panic disorder (19); association of depression and cardiovascular disease (13, 20–25); interactions of social factors and cardiovascular function (26–28); treatments for comorbid psychological and physiological conditions (29–32); and novel non-pharmacological treatment strategies for psychological and physiological disorders, such as deep brain stimulation, vagal nerve stimulation, exercise, or yoga (31, 33–35). These brief examples highlight the value of HRV research for describing and predicting several conditions that influence public health.

This integrative research topic on HRV which spans two *Frontiers* journals (*Frontiers in Medicine* and *Frontiers in Public Health*), contributes novel perspectives on the scientific and practical value of HRV research. For example, Kirby and colleagues (12) address the utility of HRV in training exercises to facilitate compassion, highlighting the importance of considering physiological processes in the context of psychological interventions. As discussed, a better understanding of behavioral, social, and emotional factors that contribute to HRV will provide evidence for its utility as an outcome measure, a potential method for manipulating behavioral and emotional states, and an index for understanding the integration of autonomic regulation and emotional reactivity (12). Similarly, Lamb et al. (35) and Steffen et al. (36) present novel findings from techniques that capitalize on altering autonomic function, including the effects of resonance frequency breathing exercises on HRV and mood in healthy humans (36), and the influence of vagal nerve stimulation on HRV and other physiological parameters in combat veterans with posttraumatic stress disorder (35). These concepts are related to those addressed by Ernst (37), who stresses the importance of considering neural–cardiac communication in the context of HRV.

To comprehensively appreciate the role that HRV measurement and manipulation has in health promotion, translation from basic to clinical research strategies is critical. To this end, the prairie vole is an example of an animal model that is used to investigate the integration of social experiences, behavior, and autonomic function (38, 39). This species is reliant on the surrounding social context for the promotion of health and behavior. Prairie voles exhibit several characteristics that mimic human social systems, including social monogamy, living in family groups, displaying biparental care of offspring, and responding negatively to social environmental disruptions (40–42). Given these characteristics, the prairie vole has been considered a useful translational model of social behavior, and the benefits of social monogamy on the endurance of this species have been discussed in the context of studies on parental behavior, pair bonding, and reproductive processes, among others (39, 42–45).

Although there is a large body of literature on prairie vole social behavior and neuroendocrine processes, research questions focused on autonomic and cardiovascular regulation using this model have only been explored over the past 10 years. This research has provided mounting evidence that the prairie vole is a valuable model for the study of social interactions and the heart (39, 46–48). The prairie vole has interesting physiological characteristics that may promote autonomic nervous system health, including a high degree of parasympathetic regulation, which in turn supports a high level of HRV (relative to other rodents, and more in line with larger mammals such as dogs and human infants) and a low resting heart rate (relative to body size scaling) (49).

Recent studies in the prairie vole model have described several autonomic correlates of behavior and physiological processes, including altered heart rate and HRV under different social conditions (50–52). This line of research has provided insight into neurobiological and behavioral processes associated with negative social experiences and stress-related disorders, including isolation, loneliness, depression, anxiety, and cardiovascular disease. For example, prairie voles exposed to social stressors display elevated heart rate, reduced HRV, impairment of endothelial-dependent vascular relaxation, cardiac arrhythmias, and cardiac gap junction protein dysregulation (38, 53–57). Some of these consequences may be due to altered neural control of peripheral processes (e.g., autonomic function, endocrine reactivity, and behavior), including changes in hypothalamic and brainstem autonomic nuclei (54, 58–61).

The issues discussed in this Frontiers research topic coupled with studies using valid and reliable animal models, will inform strategies for preventing and treating disease and facilitating good health practices. Continued dialog—such as that included in the articles focused on this HRV research topic—is crucial for ensuring that the techniques used in the laboratory are theoretically and evolutionarily grounded (37, 62–64) and are fully vetted both from a basic scientific and an applied perspective (65–68). Incorporating HRV measurement and manipulation into studies that include a translation from animal models to humans (and the reverse from human studies to the development of animal models), as well as parallel multispecies experimental protocols, will enhance our understanding of biopsychosocial factors that promote health and well-being.

## Author Contributions

AG, the sole author, was responsible for the design of this work and interpretation of findings, drafting the work, final approval of the version to be published, and is accountable for all aspects of the work to ensure its accuracy and integrity.

## Conflict of Interest Statement

This research was conducted in the absence of any commercial or financial relationships that could be construed as a potential conflict of interest.

## References

[1] World Health Organization. Constitution of WHO: Principles. (2017). Available from: http://www.who.int/about/mission/en/

[2] US Department of Health and Human Services. National Institutes of Health: Mission and Goals. (2015). Available from: https://www.nih.gov/about-nih/what-we-do/mission-goals

[3] US Department of Health and Human Services. About Healthy People. (2017). Available from: https://www.healthypeople.gov/2020/About-Healthy-People

[4] Taralov ZZ Terziyski KV Kostianev SS. Heart rate variability as a method for assessment of the autonomic nervous system and the adaptations to different physiological and pathological conditions. Folia Med (Plovdiv) (2015) 57:173–80. 10.1515/folmed-2015-0036 27180343

[5] Beauchaine TP Thayer JF. Heart rate variability as a transdiagnostic biomarker of psychopathology. Int J Psychophysiol (2015) 98:338–50. 10.1016/j.ijpsycho.2015.08.004 26272488

[6] Walker FR Pfingst K Carnevali L Sgoifo A Nalivaiko E. In the search for integrative biomarker of resilience to psychological stress. Neurosci Biobehav Rev (2017) 74:310–20. 10.1016/j.neubiorev.2016.05.003 27179452

[7] Crestani CC. Emotional stress and cardiovascular complications in animal models: a review of the influence of stress type. Front Physiol (2016) 7:251. 10.3389/fphys.2016.00251 27445843 PMC4919347

[8] Shapiro PA Sloan RP Bagiella E Bigger JT Jr Gorman JM. Heart rate reactivity and heart period variability throughout the first year after heart transplantation. Psychophysiology (1996) 33:54–62. 10.1111/j.1469-8986.1996.tb02108.x 8570795

[9] Oken BS Chamine I Wakeland W. A systems approach to stress, stressors and resilience in humans. Behav Brain Res (2015) 282:144–54. 10.1016/j.bbr.2014.12.047 25549855 PMC4323923

[10] Holzman JB Bridgett DJ. Heart rate variability indices as bio-markers of top-down self-regulatory mechanisms: a meta-analytic review. Neurosci Biobehav Rev (2017) 74:233–55. 10.1016/j.neubiorev.2016.12.032 28057463

[11] Thayer JF Ahs F Fredrickson M Sollers JJ III Wager TD. A meta-analysis of heart rate variability and neuroimaging studies: implications for heart rate variability as a marker of stress and health. Neurosci Biobehav Rev (2012) 36:747–56. 10.1016/j.neubiorev.2011.11.009 22178086

[12] Kirby JN Doty JR Petrocchi N Gilbert P. The current and future role of heart rate variability for assessing and training compassion. Front Public Health (2017) 5:40. 10.3389/fpubh.2017.00040 28337432 PMC5340770

[13] Gorman JM Sloan RP Heart rate variability in depressive and anxiety disorders. Am Heart J (2000) 140:S77–83. 10.1067/mhj.2000.109981 11011352

[14] Hamilton JL Alloy LB. Atypical reactivity of heart rate variability to stress and depression across development: systematic review of the literature and directions for future research. Clin Psychol Rev (2016) 50:67–79. 10.1016/j.cpr.2016.09.003 27697746 PMC5233715

[15] Kemp AH Quintana DS. The relationship between mental and physical health: insights from the study of heart rate variability. Int J Psychophysiol (2013) 89:288–96. 10.1016/j.ijpsycho.2013.06.018 23797149

[16] Porges SW. Social engagement and attachment: a phylogenetic perspective. Ann N Y Acad Sci (2003) 1008:31–47. 10.1196/annals.1301.004 14998870

[17] Porges SW The polyvagal theory: new insights into adaptive reactions of the autonomic nervous system. Cleve Clin J Med (2009) 76(Suppl 2):S86–S90. 10.3949/ccjm.76.s2.17 19376991 PMC3108032

[18] Zhou X Ma Z Zhang L Zhou S Wang J Wang B Heart rate variability in the prediction of survival in patients with cancer: a systematic review and meta-analysis. J Psychosom Res (2016) 89:20–5. 10.1016/j.jpsychores.2016.08.004 27663106

[19] Tucker P Adamson P Miranda R Jr Scarborough A Williams D Groff J Paroxetine increases heart rate variability in panic disorder. J Clin Psychopharmacol (1997) 17:370–6. 10.1097/00004714-199710000-00006 9315988

[20] Koenig J Kemp AH Beauchaine TP Thayer JF Kaess M Depression and resting state heart rate variability in children and adolescents – a systematic review and meta-analysis. Clin Psychol Rev (2016) 46:136–50. 10.1016/j.cpr.2016.04.013 27185312

[21] Carnevali L Montano N Statello R Sgoifo A. Rodent models of depression-cardiovascular comorbidity: bridging the known to the new. Neurosci Biobehav Rev (2017) 76:144. 10.1016/j.neubiorev.2016.11.006 28104294

[22] Sgoifo A Carnevali L Pico-Alfonso MA Amore M. Autonomic dysfunction and heart rate variability in depression. Stress (2015) 18:343–52. 10.3109/10253890.2015.1045868 26004818

[23] Carney RM Saunders RD Freedland KE Stein P Rich MW Jaffe AS. Association of depression with reduced heart rate variability in coronary artery disease. Am J Cardiol (1995) 76:562–4. 10.1016/S0002-9149(99)80155-6 7677077

[24] Glassman AH. Depression and cardiovascular comorbidity. Dialogues Clin Neurosci (2007) 9:9–17. 17506222 10.31887/DCNS.2007.9.1/ahglassman PMC3181839

[25] Kleiger RE Miller JP Bigger JT Moss AJ. Decreased heart rate variability and its association with increased mortality after acute myocardial infarction. Am J Cardiol (1987) 59:256–62. 10.1016/0002-9149(87)90795-8 3812275

[26] Cacioppo JT Hawkley LC Crawford LE Ernst JM Burleson MH Kowalewski RB Loneliness and health: potential mechanisms. Psychosom Med (2002) 64:407–17. 10.1097/00006842-200205000-00005 12021415

[27] Brunner EJ. Social factors and cardiovascular morbidity. Neurosci Biobehav Rev (2017) 74:260. 10.1016/j.neubiorev.2016.05.004 27177828 PMC5104684

[28] Wood SK Cardiac autonomic imbalance by social stress in rodents: understanding putative biomarkers. Front Psychol (2014) 5:950 10.3389/fpsyg.2014.00950 25206349 PMC4143725

[29] Roose SP Treatment of depression in patients with heart disease. Biol Psychiatry (2003) 54:262–8. 10.1016/S0006-3223(03)00320-2 12893102

[30] Chang CC Tzeng NS Yeh CB Kuo TBJ Huang SY Chang HA. Effects of depression and melatonergic antidepressant treatment alone and in combination with sedative-hypnotics on heart rate variability: implications for cardiovascular risk. World J Biol Psychiatry (2017):1–11. 10.1080/15622975.2017.1294765 28281386

[31] Toni G Belvederi Murri M Piepoli M Zanetidou S Cabassi A Squatrito S Physical exercise for late-life depression: effects on heart rate variability. Am J Geriatr Psychiatry (2016) 24:989–97. 10.1016/j.jagp.2016.08.005 27660194

[32] Kemp AH Fráquas R Brunoni AR Bittencourt MS Nunes MA Dantas EM Differential associations of specific selective serotonin reuptake inhibitors with resting-state heart rate and heart rate variability: implications for health and well-being. Psychosom Med (2016) 78:810–8. 10.1097/PSY.0000000000000336 27219492

[33] Rossi S Santarnecchi E Valenza G Ulivelli M The heart side of brain neuromodulation. Philos Trans R Soc Lond A (2016) 374(2067):1–18. 10.1098/rsta.2015.0187 27044999

[34] Tyagi A Cohen M. Yoga and heart rate variability: a comprehensive review of the literature. Int J Yoga (2016) 9:97–113. 10.4103/0973-6131.183712 27512317 PMC4959333

[35] Lamb DG Porges EC Lewis GF Williamson JB. Non-invasive vagal nerve stimulation effects on hyperarousal and autonomic state in patients with posttraumatic stress disorder and history of mild traumatic brain injury: preliminary evidence. Front Med (2017) 4:124. 10.3389/fmed.2017.00124 28824913 PMC5534856

[36] Steffen PR Austin T DeBarros A Brown T The impact of resonance frequency breathing on measures of heart rate variability, blood pressure, and mood. Front Public Health (2017) 5:222 10.3389/fpubh.2017.00222 28890890 PMC5575449

[37] Ernst G Heart rate variability – more than heart beats? Front Public Health (2017). 10.3389/fpubh.2017.00240 PMC5600971 28955705

[38] Grippo AJ Lamb DG Carter CS Porges SW. Social isolation disrupts autonomic regulation of the heart and influences negative affective behaviors. Biol Psychiatry (2007) 62:1162–70. 10.1016/j.biopsych.2007.04.011 17658486 PMC2144909

[39] Carter CS Grippo AJ Pournajafi-Nazarloo H Ruscio MG Porges SW. Oxytocin, vasopressin and sociality. Prog Brain Res (2008) 170:331–6. 10.1016/S0079-6123(08)00427-5 18655893

[40] Getz LL Carter CS Gavish L The mating system of the prairie vole, *Microtus ochrogaster*: field and laboratory evidence for pair bonding. Behav Ecol Sociobiol (1981) 8:189–94. 10.1007/BF00299829

[41] Carter CS DeVries AC Getz LL. Physiological substrates of mammalian monogamy: the prairie vole model. Neurosci Biobehav Rev (1995) 19:303–14. 10.1016/0149-7634(94)00070-H 7630584

[42] Young KA Gobrogge KL Liu Y Wang Z. The neurobiology of pair bonding: insights from a socially monogamous rodent. Front Neuroendocrinol (2011) 32:53–69. 10.1016/j.yfrne.2010.07.006 20688099 PMC3012750

[43] Aragona BJ Wang Z. The prairie vole (*Microtus ochrogaster*): an animal model for behavioral neuroendocrine research on pair bonding. ILAR J (2004) 45(1):35–45. 10.1093/ilar.45.1.35 14752206

[44] Bales KL Kim AJ Lewis-Reese AD Carter CS. Both oxytocin and vasopressin may influence alloparental behavior in male prairie voles. Horm Behav (2004) 45:354–61. 10.1016/j.yhbeh.2004.01.004 15109910

[45] Perry AN Carter CS Cushing BS Chronic social isolation enhances reproduction in the monogamous prairie vole (*Microtus ochrogaster*). Psychoneuroendocrinology (2016) 68:20–8. 10.1016/j.psyneuen.2016.02.016 26939085 PMC4851875

[46] Lewis R Curtis JT. Male prairie voles display cardiovascular dipping associated with an ultradian activity cycle. Physiol Behav (2016) 156:106–16. 10.1016/j.physbeh.2016.01.012 26780151 PMC4753128

[47] Grippo AJ. Mechanisms underlying altered mood and cardiovascular dysfunction: the value of neurobiological and behavioral research with animal models. Neurosci Biobehav Rev (2009) 33:171–80. 10.1016/j.neubiorev.2008.07.004 18703084 PMC2593749

[48] Cacioppo S Grippo AJ London S Goossens L Cacioppo JT. Loneliness: clinical import and interventions. Perspect Psychol Sci (2015) 10(2):238–49. 10.1177/1745691615570616 25866548 PMC4391342

[49] Grippo AJ Lamb DG Carter CS Porges SW. Cardiac regulation in the socially monogamous prairie vole. Physiol Behav (2007) 90:386–93. 10.1016/j.physbeh.2006.09.037 17107695 PMC1839927

[50] Kenkel WM Paredes J Lewis GF Yee JR Pournajafi-Nazarloo H Grippo AJ Autonomic substrates of the response to pups in male prairie voles. PLoS One (2010) 8(e69965):1–9. 10.1371/journal.pone.0069965 PMC3734219 23940535

[51] Kenkel WM Suboc G Carter CS. Autonomic, behavioral and neuroendocrine correlates of paternal behavior in male prairie voles. Physiol Behav (2014) 128:252–9. 10.1016/j.physbeh.2014.02.006 24534169 PMC3988699

[52] Williamson JB Lewis G Grippo AJ Lamb DG Harden E Handleman M Autonomic predictors of recovery following surgery: a comparative study. Auton Neurosci (2010) 156:60–6. 10.1016/j.autneu.2010.03.009 20451468 PMC4801019

[53] McNeal N Scotti ML Wardwell J Chandler DL Bates SL LaRocca MA Disruption of social bonds induces behavioral and physiological dysregulation in male and female prairie voles. Auton Neurosci (2014) 180:9–16. 10.1016/j.autneu.2013.10.001 24161576 PMC3947225

[54] Grippo AJ Sgoifo A Mastorci F McNeal N Trahanas DM Cardiac dysfunction and hypothalamic activation during a social crowding stressor in prairie voles. Auton Neurosci (2010) 156:44–50. 10.1016/j.autneu.2010.03.003 20347401 PMC2914185

[55] Grippo AJ Moffitt JA Sgoifo A Jepson AJ Bates SL Chandler DL The integration of depressive behaviors and cardiac dysfunction during an operational measure of depression: investigating the role of negative social experiences in an animal model. Psychosom Med (2012) 74:612–9. 10.1097/PSY.0b013e31825ca8e5 22753634 PMC3392416

[56] Peuler JD Scotti ML Phelps LE McNeal N Grippo AJ. Chronic social isolation in the prairie vole induces endothelial dysfunction: implications for depression and cardiovascular disease. Physiol Behav (2012) 106:476–84. 10.1016/j.physbeh.2012.03.019 22469565 PMC3355378

[57] Grippo AJ Moffitt JA Henry MK Firkins R Senkler J McNeal N Altered Connexin 43 and Connexin 45 protein expression in the heart as a function of social and environmental stress in the prairie vole. Stress (2015) 18:107–14. 10.3109/10253890.2014.979785 25338193 PMC4675659

[58] Grippo AJ Gerena D Huang J Kumar N Shah M Ughreja R Social isolation induces behavioral and neuroendocrine disturbances relevant to depression in female and male prairie voles. Psychoneuroendocrinology (2007) 32:966–80. 10.1016/j.psyneuen.2007.07.004 17825994 PMC2174914

[59] Yee JR Kenkel WM Frijling JL Dodhia S Onishi KG Tovar S Oxytocin promotes function coupling between paraventricular nucleus and both sympathetic and parasympathetic cardioregulatory nuclei. Horm Behav (2016) 80:82–91. 10.1016/j.yhbeh.2016.01.010 26836772 PMC5768414

[60] Bosch OJ Nair HP Ahern TH Neumann ID Young LJ The CRF system mediates increased passive stress-coping behavior following the loss of a bonded partner in a monogamous rodent. Neuropsychopharmacology (2009) 34:1406–15. 10.1038/npp.2008.154 18923404 PMC2669698

[61] Sun P Smith AS Lei K Liu Y Wang Z. Breaking bonds in male prairie vole: long-term effects on emotional and social behavior, physiology, and neurochemistry. Behav Brain Res (2014) 265:22–31. 10.1016/j.bbr.2014.02.016 24561258 PMC3983777

[62] Porges SW. The polyvagal theory: phylogenetic contributions to social behavior. Physiol Behav (2003) 79:503–13. 10.1016/S0031-9384(03)00156-2 12954445

[63] Thayer JF Lane RD Claude Bernard and the heart-brain connection: further elaboration of a model of neurovisceral integration. Neurosci Biobehav Rev (2009) 33:81–8. 10.1016/j.neubiorev.2008.08.004 18771686

[64] Smith R Thayer JF Khalsa SS Lane RD. The hierarchical basis of neurovisceral integration. Neurosci Biobehav Rev (2017) 75:274. 10.1016/j.neubiorev.2017.02.003 28188890

[65] Laborde S Mosley E Thayer JF. Heart rate variability and cardiac vagal tone in psychophysiological research – recommendations for experiment planning, data analysis, and data reporting. Front Psychol (2017) 8:213. 10.3389/fpsyg.2017.00213 28265249 PMC5316555

[66] Quintana DS Heathers JAJ. Considerations in the assessment of heart rate variability in biobehavioral research. Front Psychol (2014) 5:805. 10.3389/fpsyg.2014.00805 25101047 PMC4106423

[67] Quintana DS Alvares GA Heathers JAJ. Guidelines for reporting articles on psychiatry and heart rate variability (GRAPH): recommendations to advance research communication. Transl Psychiatry (2016) 6:1–10. 10.1038/tp.2016.73 27163204 PMC5070064

[68] Drury RL Unraveling health as a complex adaptive system: data mining, cloud computing, machine learning and biosensors as synergistic technologies. Int J Genomics Data Mining (2017) 2017(J105):1–4.

